# Self-Rated Health Associated with Chronic Diseases and Types of Physical Activity Among Middle-Aged and Older Adults: A Cross-Sectional Study Using Explainable Machine Learning

**DOI:** 10.3390/jcm15187100

**Published:** 2026-09-13

**Authors:** Tong Yang, Yongchul Choi, Yonghwan Kim, Munku Song

**Affiliations:** 1 Department of Physical Education, Kangwon National University, Gangneung Campus, Gangneung 25457, Republic of Korea; 20248068@gwnu.ac.kr (T.Y.); ycchoi@gwnu.ac.kr (Y.C.); 2 Laboratory of Integrative Physiology, Department of Health and Human Performance, University of Houston, Houston, TX 77204, USA; 3 Department of Human Healthcare, Gyeongsang National University, Jinju 52725, Republic of Korea

**Keywords:** multimorbidity, sedentary behavior, health perception, health status, KNHANES, SHAP

## Abstract

**Background**: Self-rated health (SRH) is a widely used indicator reflecting overall physical, psychological, and functional health status. Chronic diseases and physical activity (PA) are associated with SRH in middle-aged and older adults; however, the contribution of chronic disease burden and different types of PA to SRH requires further investigation. This study investigated the associations of chronic diseases and PA with SRH among middle-aged and older adults. **Methods**: This cross-sectional study included adults aged ≥40 years who participated in the 2024 Korea National Health and Nutrition Examination Survey (KNHANES), the ninth survey cycle. SRH was classified as poor or favorable based on self-reported health status. Sociodemographic characteristics, chronic diseases, and PA were evaluated using multivariable logistic regression analysis. The reported ORs were adjusted for age, sex, BMI, education level, household income, smoking, alcohol consumption, and employment status. Additional adjustment for other chronic diseases was applied in Model 3 of the overall analysis and in the subgroup analyses. Machine learning was additionally applied to evaluate the contribution of individual predictors to SRH. Model performance was evaluated using the Area Under the Receiver Operating Characteristic curve (AUROC) with five-fold cross-validation. **Results**: A total of 4079 participants were included, of whom approximately 58.1% were women, and 748 (18.3%) reported poor SRH. Participants with poor SRH were older, had lower income and education levels, and were less likely to meet the aerobic and muscle-strengthening PA guidelines than those with favorable SRH (all *p* < 0.001). All chronic diseases were significantly associated with poor SRH. Stroke (OR = 2.66, 95% CI = 1.74–4.06) and myocardial infarction or angina (OR = 2.69, 95% CI = 1.91–3.79) showed relatively high ORs, and the likelihood of poor SRH increased progressively with the number of chronic diseases. Participants with three or four chronic diseases had a 7.15-fold higher likelihood of reporting poor SRH than those without chronic diseases (95% CI = 4.63–11.04). Muscle-strengthening PA was related to lower odds of poor SRH (OR = 0.548, 95% CI: 0.435–0.690) and showed consistent inverse associations across most chronic disease groups, whereas aerobic PA showed weaker associations. The XGBoost model showed an AUROC of 0.631 based on five-fold cross-validation. SHAP analysis showed relatively greater contributions of chronic disease burden and household income to the prediction of poor SRH, while muscle-strengthening PA showed a greater contribution than the other PA variables. **Conclusions**: Chronic diseases and PA were associated with SRH among middle-aged and older adults. The likelihood of poor SRH increased with the number of chronic diseases, whereas muscle-strengthening PA was consistently associated with favorable SRH. These findings suggest that muscle-strengthening PA is associated with favorable SRH among middle-aged and older adults; however, longitudinal studies are needed to further examine this association.

## 1. Introduction

Self-rated health (SRH) is a representative subjective health indicator through which individuals evaluate their overall physical, mental, and social health status. It is also termed self-perceived health or self-assessed health [1,2]. In 2021, an average of approximately 8% of adults in Organisation for Economic Co-operation and Development (OECD) countries perceived their health status as poor [3]. Although SRH is measured using a single item, it is comprehensively affected by disease status, functional limitations, mental health, health behaviors, and socioeconomic conditions and has been widely used in behavioral and public health research to identify health inequalities and evaluate overall health status [4]. A recent study using Korean health survey data also showed that age, income, education level, and economic activity status were significantly associated with SRH [5].

Chronic diseases are important clinical determinants of SRH because they reduce physical function, increase symptom burden, and restrict participation in daily activities. Chronic diseases require long-term treatment and management and may reduce physical function and the ability to perform activities of daily living [6]. A study by Mavaddat et al. reported several meaningful findings. One-third of middle-aged and older adults aged 39 years or older had a chronic disease, and 7.5% had two chronic diseases. Compared with healthy individuals, the likelihood of poor SRH was 8.7 times higher among those with stroke and 8.5 times higher among those with a cardiac attack [7]. Multimorbidity is common among middle-aged and older adults and becomes increasingly prevalent with advancing age; a systematic review and meta-analysis reported prevalence estimates of 47.6% in populations with a mean age of 59–73 years and 67.0% in those aged ≥74 years [8]. Multimorbidity may also impose substantial physical and psychological burdens, including reduced physical function, anxiety, and difficulties in daily self-care [9]. As population ageing accelerates, identifying behavioral factors related to SRH among individuals with chronic diseases has become increasingly important.

Meanwhile, physical activity (PA) is an important modifiable health behavior that is related to SRH and plays an important role in the prevention and management of chronic diseases. Regular aerobic and muscle-strengthening PA contributes to better physical function, psychological well-being, and quality of life, whereas prolonged sedentary behavior is associated with unfavorable health outcomes [10]. A recent study reported that individuals performing at least 600 MET-minutes/week of PA were 1.8 times more likely to have favorable SRH than those with a low activity level [11]. However, according to a World Health Organization report, approximately 31% of adults worldwide did not meet the recommended PA level in 2022 [12]. Another study reported that individuals who regularly participated in aerobic and muscle-strengthening exercise had better SRH than those with insufficient PA [13].

Previous Korean studies have reported associations of SRH with socioeconomic, health-related, and behavioral factors. In a study using the 2019 KNHANES, favorable SRH was associated with higher household income, fewer unmet healthcare needs, better dietary conditions, and lower stress [5]. Another study of Korean adults with multimorbidity reported that those meeting the recommended PA level were 1.80 times more likely to report favorable SRH than those who did not meet the recommendation [14]. However, previous Korean studies have provided limited information on how individual chronic diseases, chronic disease burden, and different types of PA, including aerobic PA, muscle-strengthening PA, and sedentary behavior, are associated with SRH when examined together. Examining these factors together may provide clinically relevant information for assessing middle-aged and older adults with unfavorable perceived health and for considering their chronic disease and PA status in health assessment. Conventional regression analysis estimates the associations of individual factors with SRH but provides limited information on their relative contributions when multiple clinical, behavioral, and sociodemographic factors are considered together. Therefore, eXtreme Gradient Boosting (XGBoost) combined with SHapley Additive exPlanations (SHAP) may complement conventional analysis by evaluating the relative contribution and direction of individual predictors.

Therefore, this study investigated the associations of chronic diseases and different types of PA with SRH among Korean middle-aged and older adults using national health survey data. A machine learning approach was additionally applied to evaluate the contribution of chronic diseases, PA, and sociodemographic characteristics to SRH. We hypothesized that a greater chronic disease burden and lower levels of PA would be associated with poor SRH among middle-aged and older adults.

## 2. Methods

### 2.1. Participants

This cross-sectional study used data from the 2024 Korea National Health and Nutrition Examination Survey (KNHANES; ninth cycle), which is conducted by the Korea Disease Control and Prevention Agency. KNHANES is an annual nationwide survey that assesses the health and nutritional status of the Korean population, and participants are selected using stratified cluster probability sampling. The data are publicly available on the website for research purposes. Before participation, all participants received information about the survey and provided written informed consent. All participants voluntarily took part in the health interview, health examination, and nutrition survey and agreed that the collected data could be used for research purposes.

**Figure 1:**
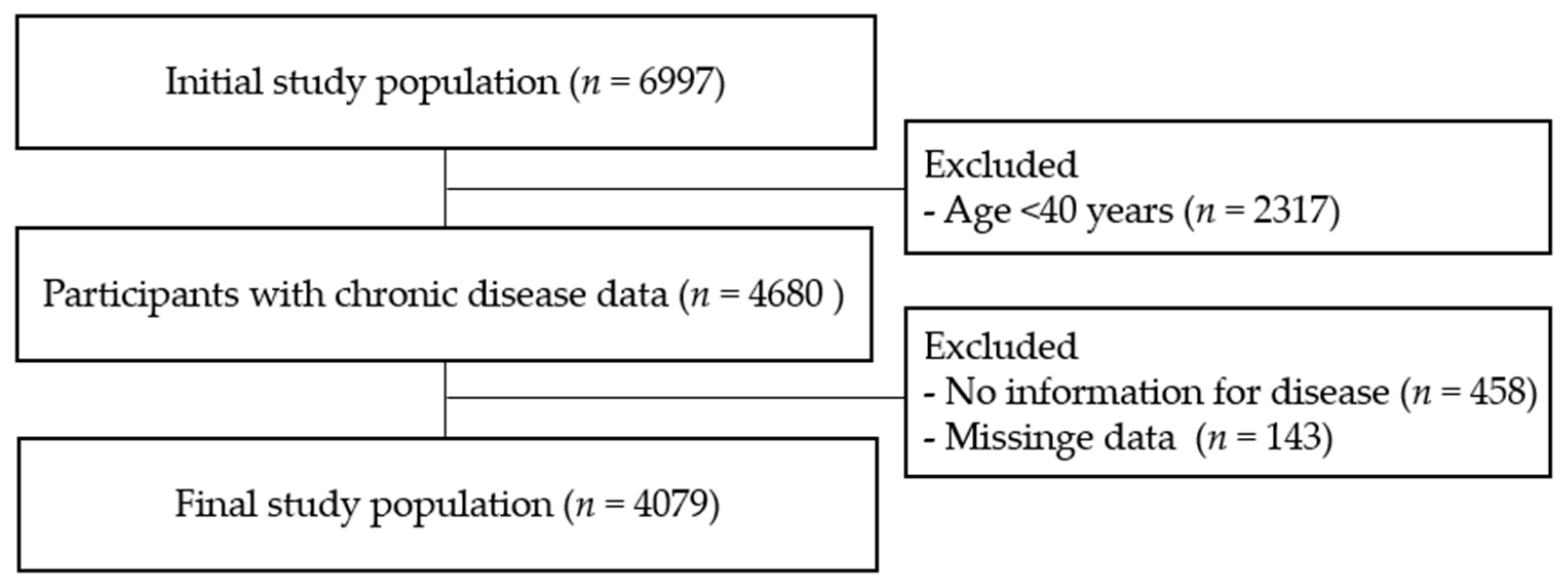
Flowchart of the study participant selection process.

This study included adults aged 40 years or older who had data on SRH, PA, chronic diseases, and socioeconomic characteristics (Figure 1). A complete-case analysis was performed. Participants with missing data for any variables included in the analysis or with data entry errors were excluded, and no imputation of missing data was performed. Because this study was a secondary analysis of the 2024 KNHANES with a predetermined survey sample, an a priori sample size calculation was not performed. All eligible participants with complete data for the variables included in the analysis were included. Finally, 4079 participants were included in the analysis. Trained investigators conducted the survey using standardized procedures. To minimize potential information bias, standardized questionnaires and assessment procedures were applied consistently to all participants. When participants had difficulty understanding or directly completing an item, the investigators provided assistance. This study obtained research ethics approval from the institution of the researchers for analysis and publication.

### 2.2. Self-Rated Health

SRH was assessed using the question, “How do you usually perceive your health?” Responses were provided on a five-point scale: very good, good, fair, poor, and very poor. In this study, very good, good, and fair were classified as favorable SRH, whereas poor and very poor were classified as poor SRH.

### 2.3. General Characteristics

General characteristics included age, sex, body mass index (BMI), monthly household income, education level, smoking, alcohol consumption, and employment status. Education level was classified as elementary school, middle or high school, or college or higher. Smoking status was classified as never, former, or current smoking according to lifetime and current smoking. Alcohol consumption was classified as no drinking, low-to-moderate-risk drinking, or high-risk drinking according to the frequency and amount of alcohol consumption based on the WHO criteria [15]. Employment status was classified as employed or not employed according to current economic activity.

### 2.4. Physical Activity

PA was assessed using the Korean version of the modified Global Physical Activity Questionnaire (GPAQ), developed by the World Health Organization (WHO), with additional questions on muscle-strengthening exercise [16]. Aerobic PA, muscle-strengthening exercise, and sedentary time were included in the analysis. The cut-off points for aerobic and muscle-strengthening PA were based on the American College of Sports Medicine recommendations for adults [17]. Participants who performed at least 150 min of moderate-intensity PA per week or at least 75 min of vigorous-intensity PA per week were considered to meet the aerobic PA guideline. The number of days during the previous week on which participants performed exercises such as push-ups, sit-ups, dumbbell exercise, weightlifting, or pull-ups was assessed. Performing muscle-strengthening exercise on at least two days per week was classified as meeting the guideline, whereas less than two days per week was classified as not meeting the guideline. Sedentary time was assessed as the average daily time spent sitting or lying down during the previous week, excluding sleep. This included sitting at work or home, transportation, reading, watching television, and computer use. The 8 h/day cut-off was selected based on the criterion suggested by Rollo et al., and sedentary time was accordingly classified as low or high [18].

### 2.5. Chronic Diseases

Among the factors investigated in KNHANES, diseases associated with high mortality were selected [19]. Cancer, myocardial infarction or angina, stroke, hypertension, and diabetes were included, and chronic disease was defined as a history of physician diagnosis. Chronic disease was calculated by summing the number of diseases in each participant. First, the presence or absence of each disease was analyzed independently. In addition, the presence of at least one chronic disease was classified as no or yes. To determine the cumulative effect according to the number of diseases, chronic disease was classified as 0, 1, 2, or 3 or more diseases. We then analyzed whether the likelihood of poor SRH increased stepwise as chronic disease increased.

### 2.6. Data Analysis

All statistical analyses were performed using IBM SPSS Statistics version 29.0 (IBM Corp., Armonk, NY, USA) and Python version 3.12 (Python Software Foundation, Wilmington, DE, USA). Statistical significance was set at *p* < 0.05. The general characteristics of the participants in Table 1 are presented as unweighted means ± standard deviations or frequencies. Poor SRH was defined as the outcome variable, while chronic diseases and PA variables were considered the primary exposure variables. Age, sex, BMI, education level, household income, smoking, alcohol consumption, and employment status were treated as potential confounders. Complex sample logistic regression analysis was performed to examine the associations between chronic diseases, PA, and poor SRH. The results are presented as odds ratios (ORs) and 95% confidence intervals (CIs). Model 1 was unadjusted. Model 2 was adjusted for age, sex, body mass index (BMI), education level, household income, smoking, alcohol consumption, and employment status. Model 3 was additionally adjusted for the other chronic diseases. To evaluate the relative importance of the predictors, an eXtreme Gradient Boosting (XGBoost (version 3.4.1)) model was developed using poor SRH as the outcome variable. SHapley Additive exPlanations (SHAP (version 0.52.0)) were subsequently applied to interpret the model and quantify the contribution of each predictor. SHAP values reflect the relative importance and contribution of variables to the model’s predictions and should not be interpreted as estimates of independent effects, causal relationships, or clinical importance. Variable importance was estimated using the mean absolute SHAP values, and SHAP summary plots were generated to visualize the relative importance of each predictor and the direction of its contribution to the predicted probability of poor SRH. Model performance was evaluated using five-fold cross-validation. Discrimination was assessed using the Area Under the Receiver Operating Characteristic curve (AUROC), and calibration was evaluated using the Brier score. No adjustment for multiple comparisons was applied because the subgroup analyses were exploratory.

## 3. Results

### 3.1. Clinical Characteristics of the Participants

A total of 748 participants (18.3%) were classified as having poor SRH. The poor SRH group was significantly older and had a higher proportion of women than the favorable SRH group (*p* < 0.001). BMI was also higher, whereas household income and education level were lower (*p* < 0.001). Alcohol consumption and employment status differed significantly between the groups, but smoking status did not differ (*p* = 0.276). The proportions meeting the aerobic and muscle-strengthening PA guidelines were significantly lower in the poor SRH group (*p* < 0.001). The prevalence of cancer, myocardial infarction or angina, stroke, hypertension, and diabetes was also significantly higher in the poor SRH group than in the favorable SRH group (*p* < 0.001).

### 3.2. Chronic Diseases and Physical Activity in Relation to Poor Self-Rated Health

Table 2 presents the results of the complex-sample logistic regression analysis of chronic diseases, PA, and poor SRH. In Model 3, which adjusted for all covariates, participants with myocardial infarction or angina and those with stroke had 2.69- and 2.66-fold higher odds of poor SRH, respectively. The odds of poor SRH progressively increased with the number of chronic diseases. Participants with three or four chronic diseases had 7.15-fold higher odds of poor SRH than those without chronic disease. Regarding PA, participants meeting the muscle-strengthening guideline and those with low sedentary time showed lower odds of poor SRH after adjustment for all covariates. The odds of poor SRH were lower among participants meeting the aerobic PA guideline in Models 1 and 2, but this difference was not significant in Model 3.

### 3.3. Poor SRH Based on Physical Activity Among Middle-Aged and Older Adults with Chronic Diseases

Table 3 presents the results for PA and poor SRH among participants with chronic diseases. Muscle-strengthening PA showed more consistent results than aerobic PA. Participants meeting the muscle-strengthening guideline had approximately 43–82% lower odds of poor SRH among those with myocardial infarction or angina, stroke, hypertension, or diabetes. Participants with low sedentary time also showed lower odds of poor SRH among those with myocardial infarction or angina, stroke, or hypertension. In contrast, aerobic PA did not show a significant OR for poor SRH in any chronic disease group.

### 3.4. Favorable SRH Based on Physical Activity Among Middle-Aged and Older Adults Without Each Specific Chronic Disease

Table 4 presents the results for PA and favorable SRH among participants without each specific chronic disease. Participants meeting the muscle-strengthening PA guideline showed higher odds of favorable SRH across all groups defined by the absence of each specific chronic disease. The odds were approximately 1.78–2.00 times higher than among those who did not meet the guideline. Participants with low sedentary time also showed higher odds of favorable SRH among those without cancer, myocardial infarction or angina, or stroke. In contrast, aerobic PA did not show a significant OR for favorable SRH in any of these groups.

### 3.5. SHAP Analysis of Predictors for Poor Self-Rated Health

**Figure 2:**
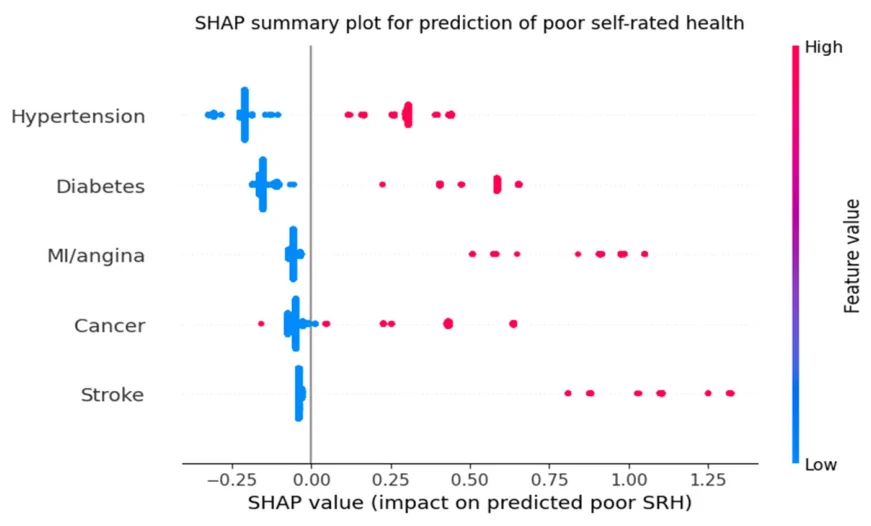
SHAP analysis of chronic disease predictors for poor SRH. The vertical line represents a SHAP value of 0, indicating no contribution to the model prediction.

Based on five-fold cross-validation, the XGBoost model yielded an AUROC of 0.631 for discrimination and a Brier score of 0.161 for calibration. Figure 2 presents the contribution of each chronic disease to the prediction of poor SRH using SHAP analysis. Stroke was the most influential variable for predicting poor SRH. The presence of stroke markedly increased the SHAP value and the predicted probability of poor SRH. This was followed by myocardial infarction or angina, diabetes, hypertension, and cancer.

**Figure 3:**
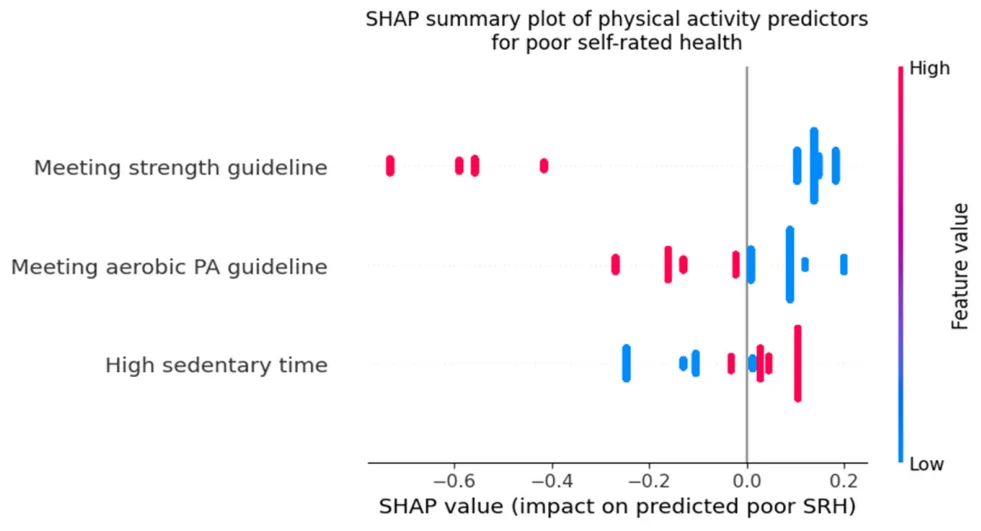
SHAP analysis of PA predictors for poor SRH. The vertical line represents a SHAP value of 0, indicating no contribution to the model prediction.

Figure 3 presents the SHAP results for the PA-related variables. Meeting the muscle-strengthening guideline was the most influential PA variable for predicting poor SRH. Meeting the guideline (feature value = high) showed negative SHAP values, indicating a lower predicted probability of poor SRH. Meeting the aerobic PA guideline also tended to reduce the predicted probability of poor SRH, but its influence was smaller than that of meeting the muscle-strengthening guideline. In contrast, high sedentary time showed positive SHAP values and increased the predicted probability of poor SRH.

**Figure 4:**
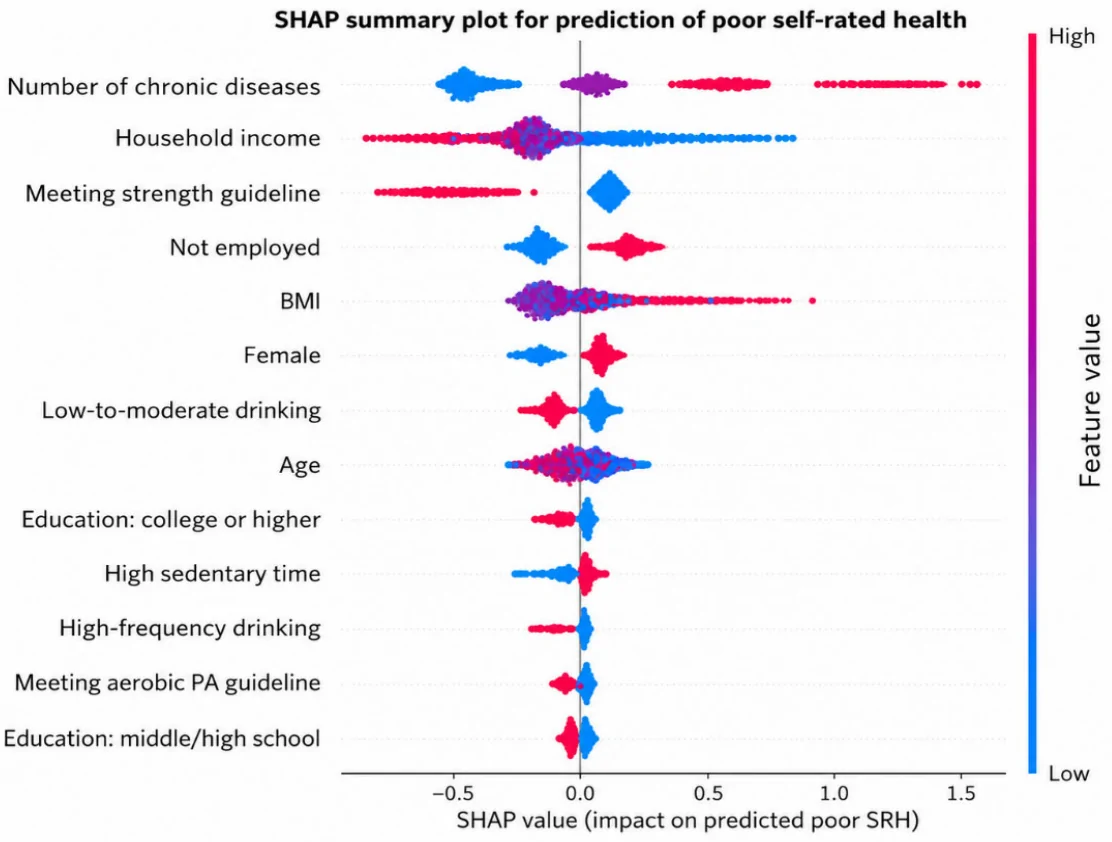
SHAP summary plot of all predictors for poor SRH. The vertical line represents a SHAP value of 0, indicating no contribution to the model prediction.

Figure 4 presents the SHAP summary plot including all predictors. The number of chronic diseases was the most influential variable for predicting poor SRH. As the number of chronic diseases increased, the SHAP value and predicted probability of poor SRH increased. Household income was the second most important variable, and lower income increased the predicted probability of poor SRH. Among the PA variables, meeting the muscle-strengthening guideline showed the highest importance and reduced the predicted probability of poor SRH. Other important variables included not being employed, BMI, female sex, low-to-moderate drinking, and age. Meeting the aerobic PA guideline and high sedentary time also contributed to the model’s predictions, but their relative importance was lower than that of meeting the muscle-strengthening guideline.

## 4. Discussion

This study examined the associations of chronic diseases and different types of PA with SRH among Korean middle-aged and older adults aged ≥40 years. The findings suggest that muscle-strengthening PA was consistently associated with favorable SRH across several chronic disease groups, whereas aerobic PA did not remain significantly associated with SRH after adjustment. The machine learning analysis provided complementary information on the contribution of chronic diseases, PA, and sociodemographic characteristics to SRH.

Muscle-strengthening PA was consistently associated with favorable SRH in this study. Among participants with chronic diseases, muscle-strengthening PA was associated with lower ORs for poor SRH, whereas among those without chronic diseases, it was associated with higher ORs for favorable SRH. The SHAP analysis also showed a relatively greater contribution of muscle-strengthening PA than aerobic PA to the prediction of SRH. These findings may be explained by the role of muscle strength in maintaining physical function and independence in middle-aged and older adults. Age-related declines in skeletal muscle mass and strength are associated with slower walking speed, an increased risk of falls, and reduced ability to perform activities of daily living [20]. This may also explain the increasing attention given to sarcopenia in ageing populations [21]. A study that measured grip strength as an indicator of muscle strength reported that older adults with weak grip strength had a 1.3-fold higher risk of functional difficulty in daily life and lower SRH [22]. A follow-up study of grip strength and SRH even reported that low grip strength and poor SRH were associated with an approximately fourfold higher risk of all-cause death than high grip strength and favorable SRH [23].

Aerobic PA was not significantly associated with SRH after full adjustment in Model 3. This finding should not be interpreted as diminishing the established health benefits of aerobic PA. Significant associations were observed in Models 1 and 2, suggesting that differences in participant characteristics and covariate adjustment may have influenced the final model. In addition, the proportion of participants meeting the aerobic PA guideline differed only modestly between the SRH groups, which may also have contributed to the nonsignificant association. Previous studies have consistently shown that aerobic PA improves cardiorespiratory fitness, reduces the risk of cardiovascular disease, and contributes to healthy ageing [24]. A meta-analysis of 42 studies reported that aerobic fitness reduced all-cause and cardiovascular disease mortality by 14% and 16%, respectively [25]. These findings indicate that aerobic and muscle-strengthening PA should be considered separately when examining their relationships with SRH.

Sedentary behavior was also associated with SRH in this study. Unlike physical inactivity, sedentary behavior refers to prolonged sitting or reclining during waking hours and may occur even among individuals who meet the recommended PA guidelines [26]. Increasing evidence suggests that reducing sedentary behavior provides additional health benefits beyond increasing PA alone [27]. In this study, participants with low sedentary time had lower ORs for poor SRH, and high sedentary time increased the predicted probability of poor SRH in the SHAP analysis. Although PA has been widely studied, relatively fewer studies have examined sedentary behavior, which is generally not beneficial to health. The quartile with the longest sedentary time had up to a 2.6-fold higher relative risk of mortality [28], and depression was reported to increase by up to 1.6 times among individuals with long sedentary time [29].

All chronic diseases were significantly associated with poor SRH, but myocardial infarction or angina and stroke showed the highest ORs. These results may be explained by the physical disability and reduced quality of life caused by cardiovascular and cerebrovascular diseases. Many studies have reported that stroke survivors often experience reduced walking ability, limited activities of daily living, and depression. These difficulties may lead them to perceive their health status negatively [30,31]. Patients with ischemic heart disease have also been reported to have poor health-related quality of life because of reduced exercise capacity, persistent fatigue, anxiety about recurrence, and psychological stress [32,33]. In the present SHAP analysis, stroke and myocardial infarction or angina also showed the highest variable importance, consistent with the logistic regression results. Therefore, the stronger associations of cardiovascular and cerebrovascular diseases with poor SRH than those of other chronic diseases can be explained by the characteristics of SRH, which reflects both functional limitations and psychological burden in addition to the disease itself.

Previous studies have also reported poorer SRH among individuals with cancer, hypertension, and diabetes [34]. Consistent with these findings, cancer, hypertension, and diabetes showed significant relationships with poor SRH after adjustment for covariates in the present study. Cancer may be accompanied by persistent symptoms such as fatigue and by treatment-related burden, which can affect physical functioning and perceived health [35]. Hypertension typically requires long-term monitoring and treatment and frequently coexists with other chronic conditions, increasing the complexity of disease management [36]. Diabetes requires continuous self-management and may be accompanied by complications and psychosocial burden that can affect daily functioning and perceived health [37]. These disease-specific characteristics may partly explain the observed associations with poor SRH. Sociodemographic differences were also observed, particularly for age, sex, household income, education, and employment status. Previous Korean studies have similarly shown that SRH varies according to socioeconomic characteristics, with income and educational attainment contributing substantially to socioeconomic inequalities in SRH [5,38]. These findings indicate that SRH may reflect not only chronic disease burden but also the broader socioeconomic circumstances of middle-aged and older adults.

The number of chronic diseases was an important factor explaining poor SRH in this study. SHAP values reflect contributions to the model’s predictions rather than independent, causal, or clinical effects. Multimorbidity is recognized as an important public health problem in an aging society and results in both increased medical costs and reduced quality of life [39]. Interactions among diseases may further worsen physical function and mental health and negatively affect SRH. Similarly, a cross-sectional study by Honda et al. reported that individuals with multimorbidity had a 30% higher likelihood of selecting poor SRH [40], and a longitudinal cohort study showed a 1.9-fold higher risk of reporting poor SRH among those with multimorbidity [41]. These findings explain why multimorbidity requires more careful management than a single chronic disease in middle-aged and older adults. The SHAP summary plot in Figure 4 presents not only the relative importance of individual predictors but also the direction of their contributions to the prediction of poor SRH.

The significance of this study lies in the association of chronic disease burden with poor SRH among Korean middle-aged and older adults, with myocardial infarction or angina and stroke showing relatively strong associations among the individual diseases. Muscle-strengthening PA also showed a more consistent inverse association with poor SRH than aerobic PA and contributed to the prediction of SRH in the SHAP analysis. These findings suggest that muscle-strengthening exercise and chronic disease management may be relevant to favorable SRH among individuals. This study also applied XGBoost and SHAP analyses to complement the conventional regression results and evaluate the relative contribution of individual variables to the model’s predictions. Given the AUROC of 0.631, the model has limited discriminative ability and is better suited for exploratory interpretation than for individual clinical application. Explainable artificial intelligence has been increasingly applied in public health not only for disease prediction but also to support the interpretation of multiple health-related factors and inform health management strategies. The present study provides an example of the application of this approach to SRH among individuals [42].

The clinical implications of these findings are as follows. SRH is a simple measure that may provide clinicians with additional information on an individual’s perceived health status alongside the assessment of chronic diseases. Those with multiple chronic diseases or unfavorable SRH may benefit from further assessment of PA and functional status. The observed relationships with muscle-strengthening PA and sedentary time suggest that these behaviors may also be considered when evaluating overall health status. These findings suggest that SRH may complement chronic disease and PA assessments in providing a more comprehensive view of health status among middle-aged and older adults.

This study has several limitations. First, because this was a cross-sectional study, causal relationships between chronic diseases, PA, and poor SRH could not be determined. Reverse causality is also possible, as poor SRH or functional limitations associated with chronic diseases may reduce participation in PA. In particular, the significant association of muscle-strengthening PA and the nonsignificant association of aerobic PA do not indicate that muscle-strengthening PA has a greater role in SRH. Second, SRH and PA were assessed using self-reported questionnaires; therefore, recall bias and social desirability bias cannot be excluded. Third, PA was classified according to whether the recommended guidelines were met and did not fully reflect exercise intensity, frequency, and duration. Fourth, the complete-case approach excluded participants with missing data, which may have introduced selection bias and affected the representativeness of the study sample. Fifth, although five-fold cross-validation was used for internal validation of the XGBoost model, external validation was not performed; therefore, its generalizability to other populations remains uncertain. Finally, potential confounding variables such as depression, cognitive function, and social support were not included.

## 5. Conclusions

This study showed that chronic diseases and PA were associated with SRH among Korean middle-aged and older adults. The odds of poor SRH increased with the number of chronic diseases, whereas participants meeting the muscle-strengthening PA guideline showed more favorable SRH patterns across several chronic disease groups. In addition, the machine learning analysis provided complementary information on the contribution of chronic diseases, PA, and sociodemographic characteristics to the model’s predictions of SRH. These findings indicate that chronic disease burden and PA are relevant factors in SRH; however, causal relationships cannot be inferred from this cross-sectional study. Future longitudinal studies should examine whether changes in chronic disease burden and specific types of PA, including aerobic, muscle-strengthening, and combined aerobic and muscle-strengthening activities, predict subsequent changes in SRH.

## Figures and Tables

**Table 1:** Clinical characteristics of participants according to self-rated health status.

Variables	Favorable SRH (n = 3331)	Poor SRH (n = 748)	ES	*t* or *x*^2^	*p*
Age, years	60.2 ± 11.5	63.6 ± 11.7	0.295	−7.348	<0.001
Sex, Male/Female	43.5%/56.5%	34.8%/65.2%	0.069	19.172	<0.001
Body mass index, kg/m^2^	24.1 ± 3.4	24.8 ± 4.1	0.198	−3.918	<0.001
Income, KW, thousands	556.9 ± 386.8	428.2 ± 359.0	0.337	8.734	<0.001
Education					
Elementary school	499 (15.0%)	217 (29.0%)	0.154	96.313	<0.001
Middle and high school	1508 (45.3%)	331 (44.3%)			
College or higher	1324 (39.7%)	200 (26.7%)			
Smoking					
Never smoker	2055 (61.7%)	485 (64.8%)	0.025	2.576	0.276
Former smoker	755 (22.7%)	156 (20.9%)			
Current smoker	521 (15.6%)	107 (14.3%)			
Alcohol					
Non drinker	1026 (30.8%)	337 (45.1%)	0.118	56.414	<0.001
Low and medium risk	1651 (49.6%)	303 (40.5%)			
High risk	654 (19.6%)	108 (14.4%)			
Occupation					
Yes	2139 (64.2%)	353 (47.2%)	0.135	74.464	<0.001
No	1192 (35.8%)	395 (52.8%)			
Physical activity					
Aerobic, Yes	1326 (39.8%)	240 (32.1%)	0.061	15.401	<0.001
Strength, Yes	883 (26.5%)	109 (14.6%)	0.108	47.285	<0.001
Disease, Yes (%)					
Cancer	252 (7.6%)	93 (12.4%)	0.068	18.694	<0.001
MI or angina	103 (3.1%)	71 (9.5%)	0.165	61.262	<0.001
Stroke	58 (1.7%)	46 (6.1%)	0.108	47.780	<0.001
Hypertension	1090 (32.7%)	377 (50.4%)	0.143	82.890	<0.001
Diabetes	466 (14.0%)	216 (28.9%)	0.154	97.225	<0.001

*p* < 0.05; MI, myocardial infarction; KW, Korean Won; ES, effect size.

**Table 2:** Odds ratio of poor self-rated health based on chronic disease and physical activity.

Variables	Disease	Model 1 OR (95% CI)	Model 2 OR (95% CI)	Model 3 OR (95% CI)
Chronic disease				
Cancer	YES	1.735 (1.348–2.233)	1.504 (1.157–1.957)	1.563 (1.195–2.403)
MI, angina	YES	3.287 (2.402–4.497)	2.771 (1.981–3.876)	2.691 (1.910–3.791)
Stroke	YES	3.698 (2.490–5.491)	2.833 (1.871–4.290)	2.660 (1.740–4.064)
Hypertension	YES	2.089 (1.779–2.453)	1.684 (1.399–2.027)	1.511 (1.248–1.828)
Diabetes	YES	2.496 (2.073–3.006)	2.078 (1.705–2.533)	1.899 (1.549–2.327)
≥1 disease vs. 0 diseases	-	2.546 (2.154–3.009)	2.098 (1.730–2.544)	-
Number disease, n	0	reference	reference	
	1	1.925 (1.592–2.324)	1.675 (1.356–2.068)	
	2	3.405 (2.727–4.252)	2.934 (2.283–3.772)	
	3–4	8.683 (5.835–12.923)	7.145 (4.626–11.036)	
Physical activity				
Aerobic PA	YES	0.714 (0.604–0.845)	0.831 (0.697–0.990)	0.841 (0.703–1.005)
Strength PA	YES	0.473 (0.381–0.588)	0.530 (0.423–0.663)	0.548 (0.435–0.690)
Sedentary time, <8 h	YES	0.748 (0.630–0.888)	0.765 (0.642–0.911)	0.806 (0.672–0.967)

*p* < 0.05; PA, physical activity; MI, myocardial infarction; OR, odds ratio; CI, confidence interval. Model 1: Crude OR; Model 2: age, sex, body mass index, income, education, alcohol, occupation, physical activity; Model 3: Model 2 + other chronic diseases.

**Table 3:** Physical activity and poor SRH among middle-aged and older adults with chronic diseases.

Variables	PA	Aerobic PA OR (95% CI)	Strength PA OR (95% CI)	Sedentary Time OR (95% CI)
With cancer (n = 345)	not meeting guideline	reference	reference	reference
	meeting guideline	0.926 (0.539–1.592)	0.990 (0.521–1.880)	0.939 (0.540–1.632)
With MI, angina (n = 174)	not meeting guideline	reference	reference	reference
	meeting guideline	0.782 (0.241–2.389)	0.369 (0.158–0.860)	0.418 (0.192–0.909)
With stroke (n = 104)	not meeting guideline	reference	reference	reference
	meeting guideline	0.415 (0.149–1.150)	0.178 (0.046–0.697)	0.338 (0.124–0.923)
With hypertension (n = 1467)	not meeting guideline	reference	reference	reference
	meeting guideline	0.899 (0.682–1.185)	0.549 (0.387–0.780)	0.667 (0.506–0.881)
With diabetes (n = 682)	not meeting guideline	reference	reference	reference
	meeting guideline	0.976 (0.675–1.412)	0.569 (0.357–0.908)	0.704 (0.484–1.024)

*p* < 0.05; PA, physical activity; MI, myocardial infarction; OR, odds ratio; CI, confidence interval. ORs were adjusted for age, sex, BMI, education level, household income, smoking, alcohol consumption, employment status, and other chronic diseases, as appropriate.

**Table 4:** Physical Activity and Favorable SRH among Middle-Aged and Older Adults without Each Specific Chronic Disease.

Variables	PA	Aerobic PA OR (95% CI)	Strength PA OR (95% CI)	Sedentary Time OR (95% CI)
Without cancer (n = 3734)	not meeting guideline	reference	reference	reference
	meeting guideline	1.190 (0.989–1.437)	2.003 (1.568–2.560)	1.490 (1.232–1.797)
Without MI and angina (n = 3905)	not meeting guideline	reference	reference	reference
	meeting guideline	1.198 (0.998–1.438)	1.816 (1.434–2.299)	1.202 (1.001–1.443)
Without stroke (n = 3975)	not meeting guideline	reference	reference	reference
	meeting guideline	1.164 (0.973–1.393)	1.780 (1.415–2.241)	1.213 (1.013–1.453)
Without hypertension (n = 2612)	not meeting guideline	reference	reference	reference
	meeting guideline	0.819 (0.648–1.037)	1.850 (1.364–2.511)	1.101 (0.869–1.395)
Without diabetes (n = 3397)	not meeting guideline	reference	reference	reference
	meeting guideline	1.205 (0.983–1.478)	1.852 (1.424–2.409)	1.200 (0.979–1.471)

*p* < 0.05; PA, physical activity; MI, myocardial infarction; OR, odds ratio; CI, confidence interval. ORs were adjusted for age, sex, BMI, education level, household income, smoking, alcohol consumption, employment status, and other chronic diseases, as appropriate.

## Data Availability

Publicly available datasets were analyzed in this study. The data presented in this study are openly available in the Korea National Health and Nutrition Examination Survey database at https://knhanes.kdca.go.kr (accessed on 6 June 2026).
